# Cervical Cancer Screening Uptake Among Women with Disabilities: Findings from a Cross-Sectional Study in Chile

**DOI:** 10.3390/ijerph21121578

**Published:** 2024-11-27

**Authors:** Sergio Jara-Rosales, Elena S. Rotarou

**Affiliations:** 1 Faculty of Health Care Sciences, School of Midwifery, Universidad San Sebastián, Los Leones Campus, Santiago 7510157, Chile; sergio.jara@uss.cl; 2 Doctorate Program in Chronic Diseases, Faculty of Medicine and Science, Universidad San Sebastián, Los Leones Campus, Santiago 7510157, Chile; 3 ANID Millennium Science Initiative Program, Millennium Nucleus Disability and Citizenship (DISCA), Project No. NCS2022_039, Santiago 7510157, Chile; 4 Department of Public Health, Faculty of Medicine and Science, Universidad San Sebastián, Los Leones Campus, Santiago 7510157, Chile

**Keywords:** cervical cancer, equity, Papanicolaou test, screening, women with disabilities

## Abstract

The Papanicolaou (Pap) test is one of the most effective methods for cervical cancer screening. However, women with disabilities are less likely to be screened. The objective of this study is to determine whether there is a difference in Pap test utilisation between women with and without disabilities and to analyse the demographic, socioeconomic, and health-related characteristics associated with Pap test uptake among women with disabilities in Chile. Data from the 2022 National Socioeconomic Characterization Survey were analysed, and 71,989 women between 18 and 75 years of age were included. The dependent variable was Pap test utilisation, and the exposure variable was disability. We used logistic regressions to control for demographic, socioeconomic, and health-related covariates. The results showed that women with disabilities were less likely to undergo a Pap test compared to those without disabilities. Characteristics such as being married, being aged between 25 and 64 years, and having secondary or tertiary education increased the likelihood of Pap test utilisation. Conversely, being single, having received special education, and being inactive decreased these odds. Effective public health policies are needed that will increase Pap test utilisation for all population subgroups, including women with disabilities and, therefore, promote health equity.

## 1. Introduction

Epidemiological and socioeconomic changes have resulted in an increase in the prevalence of chronic diseases and, therefore, have stressed the need for the adoption and implementation of effective policies that will improve health outcomes. Despite the promotion of preventive health services as one of the most valuable tools for the early detection of diseases, access to such services is not equitable. The COVID-19 pandemic has aggravated existing health inequities, with various studies showing lower rates of preventive exam utilisation, when compared to pre-pandemic data, not only for the general population [1,2] but also for disadvantaged subpopulation groups [3,4], such as women with disabilities.

Most of the existing evidence suggests that women with disabilities have greater health needs and may present higher utilisation rates but worse access to health services in general, and preventive services in particular, both pre- and post-COVID-19 pandemic [5,6,7,8,9,10]. A systematic review regarding general health care among people with disabilities in Latin America and the Caribbean revealed that people with disabilities use more healthcare services than those without disabilities [11]. Studies have shown that women with disabilities were less likely to receive cancer screening due to barriers such as lack of information, transportation difficulties, non-adapted equipment and facilities, communication challenges, and negative attitudes from healthcare professionals, highlighting the health inequities faced by this population subgroup [12,13,14].

Cervical cancer is the fourth most common cancer in women in the world, after breast, lung, and colorectum cancer. In 2022, the age-adjusted global incidence was 14.1 per 100,000 people, and mortality was 7.1 per 100,000 people [15]. These figures vary by region and are related to countries’ level of economic development and their ability to implement comprehensive cervical cancer programmes [16,17]. Countries with a low human development index, such as countries in East Africa (40.4 and 28.9 per 100,000 people, respectively), Southeast Asia (17.4 and 9.5 per 100,000 people, correspondingly), and South America (15.6 and 7.8 per 100,000 people, respectively), present the highest cervical cancer incidence and mortality [15,17,18,19]. On the contrary, incidence rates in regions with high-income countries, such as North America (6.1 per 100,000 people), Australia and New Zealand (5.6 per 100,000 people), and Western Asia (4.1 per 100,000 people), are between 7 and 10 times lower compared to transition economies [15,18,19].

In the case of Latin America, cervical cancer constitutes an important public health problem. Despite a decrease in the incidence and mortality rates observed in the last decade in women older than 30 years of age, the rates continue to be below the target elimination rate proposed by the World Health Organisation (WHO) of less than four cases per 100,000 women [16,18,20]. In 2022, the incidence rate of cervical cancer in Chile was 11.3 per 100,000 people, which is a rate lower than the average incidence rate in South America but higher than the average rate in high-income countries, with this type of cancer being the fifth cause of death in Chilean women [21].

The primary screening tests for adult women that can help prevent cervical cancer or detect it early are the Papanicolaou (Pap) test, which identifies precancerous cell changes in the cervix, and the human papillomavirus (HPV) test, which detects the virus that may cause such changes. The HPV test can be performed alone (primary HPV test) or with the Pap test (co-testing) [22,23]. Screening recommendations vary based on patient characteristics and national guidelines. In general, the US Centers for Disease Control and Prevention (CDC) guidelines suggest that women aged 21–29 undergo a Pap test every three years, while women aged 30–65 may opt for a Pap test every three years, an HPV test every five years, or both tests every five years [24].

Taking into account that screening programmes—particularly the Papanicolaou test and the human papillomavirus (HPV) test—have been shown to reduce cervical cancer rates between 50% and 80%, inequity in test utilisation by women with disabilities has important effects on morbidity and mortality rates from cervical cancer [25]. Persisting inequity can also lead to a failure to achieve the World Health Organization’s (WHO) ‘90–70–90’ plan, that is 90% vaccination against the human papillomavirus (HPV), 70% coverage (screening), and 90% treatment [20]. The achievement of Sustainable Development Goal 3, and particularly Target 3.8, that is, achieve universal health coverage, including financial risk protection, access to quality essential healthcare services and access to safe, effective, quality, and affordable essential medicines and vaccines for all, can be seriously undermined as well by the existing inequity in preventive exams utilisation.

Considering that one of the main objectives of health systems is the reduction in health inequities, it is important that population-level data are available concerning which factors may affect the utilisation of preventive services by women with disabilities so that policies and targeted interventions can be implemented. This topic is very relevant considering that the United Nations Convention on the Rights of Persons with Disabilities (UNCRPD), which Chile signed in 2007, recognises as part of the human rights of persons with disabilities the right to receive equitable treatment in access to health care [26]. Bearing in mind the reasons mentioned above, it is very relevant for the health and well-being of women with disabilities, but also for society and health systems, to have population-level data regarding the potential factors affecting the utilisation of preventive services by women with disabilities, in order to be able to implement strategies that improve their access to the available screening tests [27].

The objective of this study is two-fold: (a) to determine whether there is a difference in the utilisation of the Papanicolaou test between women with and without disabilities in Chile in 2022 and (b) to identify the potential demographic, socioeconomic, and health-related factors associated with the undertaking of the Papanicolaou test by women with disabilities. To the best of our knowledge, the current article is the only one available on Pap test utilisation for women with disabilities using population data post-COVID-19 not only in Chile but also in an international context. The very few international studies available on the topic published post-COVID-19 examine pre-pandemic data (for example, Baruch et al. [28] on women with physical disability).

### Cervical Cancer and Women with Disabilities in Chile

In Chile, the cervical cancer screening program was implemented in the early 1970s. However, between 1970 and 1985, it did not have a significant impact on mortality rates. It was not until 1990, following improvements to the program, that a decrease in mortality was observed [29]. From 1990 to 2003, there was a sustained and significant downward trend in the age-adjusted mortality rate for cervical cancer, declining from 14.3 per 100,000 women in 1990 to 8.5 per 100,000 women in 2003 [30]. By 2012, the mortality rate had decreased to 5.5 per 100,000 women, representing a 67.2% reduction in mortality between 1990 and 2012 [31]. According to the International Agency for Research on Cancer, the age-adjusted cervical cancer mortality rate in 2022 was 5.2 per 100,000 women, continuing the downward trend [21].

Since 2005, the Pap test has been included as part of the Explicit Health Guarantees (GES) program for cervical cancer, ensuring that all women aged 25 to 65 years have access to the Pap test every 3 years, regardless of their health insurance coverage [27]. The GES program covers individuals aged 15 and older and includes screening/suspicion (abnormal Pap), diagnosis, treatment, and follow-up, encompassing medications, supplies, surgery, and tests. The amount to be paid depends on the health insurance provider. Individuals under the National Health Fund (FONASA) receive free care in the public health system, while those with private insurance (ISAPREs) pay 20% of the total cost [32]. The HPV test is not covered by the GES program; women who undertake it have to pay for it out of pocket.

Concerning disability, there are 2,703,893 adults in Chile—that is, 17.6% of the population—that experience some type of disability, with more women compared to men with disabilities (21.9% and 13.1%, respectively) [33], evidencing the gender dimension of disability. The 21.9% percentage of women with disabilities is composed of 14.6% of women with severe disability and 7.3% of women with mild or moderate disability [33]. In relation to access to health services, there are few existing national studies that generally reveal worse access to health services by people with disabilities [34,35,36] and worse experience in accessing health services by women with disabilities, mainly due to physical, attitudinal, and communication barriers [37]. There are very few studies on the utilisation of screening services for cancer prevention in women with disabilities [38,39].

## 2. Materials and Methods

### 2.1. Data

This study employed data from the 2022 National Socioeconomic Characterisation Survey (CASEN), which is a survey conducted every two to three years since 1990 by the Ministry of Social Development and Family of the Government of Chile. The aim of the CASEN is to estimate the magnitude of poverty and income distribution in the country, as well as to evaluate the impact of social policies, with a particular focus on priority groups, such as children and adolescents, young people, older people, women, indigenous people, and people with disabilities. The 2022 survey also aimed at providing key data for the country’s economic and social recovery process after the COVID-19 pandemic. For the 2022 CASEN, the executing agencies were (a) the National Institute of Statistics (sample design and development of expansion factors); (b) the Centre for Microdata at the University of Chile (data collection and processing); and (c) Cadem (external supervision of training, fieldwork, and data collection) [40].

The general coverage of the 2022 CASEN is the population living in private households and residing in the territory of the country. The sampling frame is based on the 2020 Housing Sampling Frame, which, in turn, is based on information from the 2017 Population and Housing Census, updated to 2020. This frame consists of primary sampling units (PSUs), which correspond to geographic areas that are homogeneous in terms of the number of occupied private dwellings they contain, excluding seasonal and collective housing. The sampling method was probabilistic, stratified, and in two stages, with national and regional representativeness. The sampling strata were defined by the combination of municipality–area–socioeconomic level. The PSUs are clusters of households, and the final selection unit is the household [40].

The units of analysis were people and households. The 2022 survey reached a sample size of 72,056 households, with a total of 86,114 family nuclei, and 202,231 people were identified and characterised. A detailed presentation of the complex sampling method and calculation of the sampling size can be found in the 2022 CASEN “Methodology of sampling design” document [41].

The interviews with adult residents were performed using the CAPI modality (computer-assisted personal interview), with the help of mobile devices (tablets), and by employing the Survey Solutions 22.06 software, developed by the World Bank for information collection. The survey was conducted for the first time using tablets, which were provided by the Centre for Microdata at the University of Chile to each of the more than 1100 interviewers [42,43]. The interviews were undertaken during the period from 1 November 2022 to 2 February 2023 and lasted approximately one hour and five min (for a family of four). They were voluntary, and no personal data were required (such as ID number or last name). As the database is freely available to the public on the website of the Ministry of Social Development and Family [40] and all data are anonymous, ethical approval was not required for the current research (letter of exemption from the Ethics Scientific Committee of the researchers’ university is available upon request). The authors confirm that all procedures and analyses in this work comply with the ethical standards of the relevant national committees regarding research on humans and with the Helsinki Declaration of 1975, as revised in 2008.

The questionnaire included eight thematic modules, with a total of 611 variables, namely (1) resident registration; (2) education; (3) work; (4) income; (5) health; (6) identities, networks, and participation; (7) housing; and (8) sexual orientation and gender identity [44]. The following additional variables were also included: (a) household identification (17 variables); (b) survey weights (4 variables); (c) income-related variables generated by the UN Economic Commission for Latin America and the Caribbean (220 variables); (d) income-related variables generated by the Ministry of Social Development and Family (12 variables); (e) multidimensional poverty variables (25 variables); and (f) other variables generated by the Ministry of Social Development and Family (28 variables) [45].

We adopted the Strengthening the Reporting of Observational Studies in Epidemiology (STROBE) guidelines [46] (Table S1, Supplementary Material).

### 2.2. Variables

The dependent variable “Undertaking of a Pap test” was constructed based on the question “In the last three years, have you had a Papanicolaou test?”, taken from the health module of the CASEN survey. This qualitative variable was binary (yes/no). There is no question in the survey regarding the uptake of the VPH test.

The independent variable “disability” was built based on the binary variable “Do not have any of [the above] conditions of long duration”, from the health module of the CASEN survey. The conditions of long duration specified were (a) physical and/or mobility difficulty; (b) muteness or speech difficulty; (c) psychiatric difficulty; (d) mental or intellectual difficulty; (e) deafness or difficulty in hearing; and (f) blindness or difficulty in seeing. In the CASEN survey, disability was self-reported and defined according to the United Nations Convention on the Rights of Persons with Disabilities, which specified that persons with disabilities “…include those who have long-term physical, mental, intellectual or sensory impairments which in interaction with various barriers may hinder their full and effective participation in society on an equal basis with others” [47].

Other independent demographic, socioeconomic, and health-related variables used in this study included the following:

1. Age groups: 18–24/25–34/35–49/50–64/65–75. Survey question: What is your age?
2. Civil status: married/living with or in a relationship/separated, divorced, annulled/widowed/single. Survey question: What is your marital or civil status?
3. Zone: urban/rural. This information was provided by the trained interviewer, depending on the location of the private home;
4. Education: did not attend school/special/primary/secondary/tertiary. Survey question: What is the highest educational level that you achieved?
5. Employment: employed/unemployed/inactive (‘inactive’ people are those who do not work, and neither do they seek employment). This variable was generated by the Ministry of Social Development and Family, based on a number of employment-related variables;
6. Per capita family income: 0–350,000/350,001–500,000/500,001–1,000,000/1,000,001+ Chilean pesos (CHP). This variable was generated by the UN Economic Commission for Latin America and the Caribbean based on a number of income-related variables;
7. Health insurance: FONASA (public)/ISAPRE (private)/armed forces and order/none (out-of-pocket). Survey question: To what health insurance system are you affiliated?
8. Medical treatment: No/Yes (question on whether people receive treatment for at least one out of 22 diseases, such as hypertension, diabetes, depression, and various types of cancer). Survey question: In the last 12 months, have you been in medical treatment for any of the following diseases?

The inclusion criteria of the current study were (1) women between 18 and 75 years of age and (2) women who answered the question “In the last three years, have you had a Papanicolaou test?” from the 2022 CASEN survey. We included women outside the target age for screening as well due to the fact that, on the one hand, research has shown that the mean age at first Pap test is getting lower and that a significant percentage of women below 25 years of age are undergoing the test [48], and on the other hand, that more women over 65 years of age continue to get screened, as evidence has shown the benefits of undertaking the Pap test even after the cut-off age and the need to review the guidelines [49].

There were a few missing values (6.1% for the Pap test variable, 0.7% for the health insurance variable, and 1.1% for the medical treatment variable). Case deletion (default in Stata), which analyses cases with available data on each variable, was minimal. Since we have a large sample, the statistical power is considered sufficiently high [50]. With regards to the question on the Pap test, 6.3% of women without disabilities answered that they did not know about the test or did not remember having conducted it or not. For women with disabilities, this percentage was 4.0%. There was no statistically significant difference between women who replied to this question and those who did not, with regard to income. Concerning age, about 75% of the women who did not reply were within the target group age for the test; this percentage was 75.7% for the women who replied to this question. With regards to education, 87% of the women who did not reply had secondary and tertiary education, a percentage that is slightly higher than the equivalent of the women who did respond (82.8%).

The sample size of this study included 71,989 adult women, of which 11,041 (15.3%) were women with disabilities and 60,948 (84.7%) were women without disabilities.

### 2.3. Statistical Analysis

In order to determine the demographic, socioeconomic, and health-related differences between women with or without disabilities, chi-square tests were applied. Multivariable logistic regressions were used to determine if there is a difference in the probability of Pap test utilisation between women with and without disabilities (Objective 1) and to establish the association between various potential factors and Pap test utilisation by women with disabilities (Objective 2). Estimated probabilities were also calculated for the utilisation of the Papanicolaou test, with ‘age groups’ and ‘education’ as the predictor variables.

Due to the survey’s complex sample design, survey weights and probability weights were used; all analyses in the study are weighted to reflect population estimates.

All statistical analyses were performed using the STATA©15 programme.

## 3. Results

Table 1 summarises the characteristics of the study sample. Regarding the Pap test, there are more women without disabilities who underwent the test (61%) compared to women with disabilities (54.5%); this difference is statistically significant. Women with disabilities in the 25–64 age range (the target age range for undertaking the Pap test) constituted 65.2% of all women with disabilities in the sample, while women without disabilities in the same age range made up 76.7%. Concerning civil status, among women with disabilities, 35.4% were married (vs. 31.3% of women without disabilities), and 27.2% were single (vs. 32.6% of women without disabilities).

Regarding education, a marked difference is observed between the two groups, with 31.5% of women with disabilities having completed only primary education, compared to 14.0% in the group without disabilities. Meanwhile, 43.7% of women without disabilities had completed tertiary education, compared to 23.9% of women with disabilities. In relation to employment, the percentage of inactive women is much higher in the group of women with disabilities (60.9%), compared to the group of women without disabilities (39.5%). Finally, there was a much higher percentage of women with disabilities undergoing medical treatment for a health condition (72.1%) in relation to women without disabilities (34.3%).

**Figure 1:**
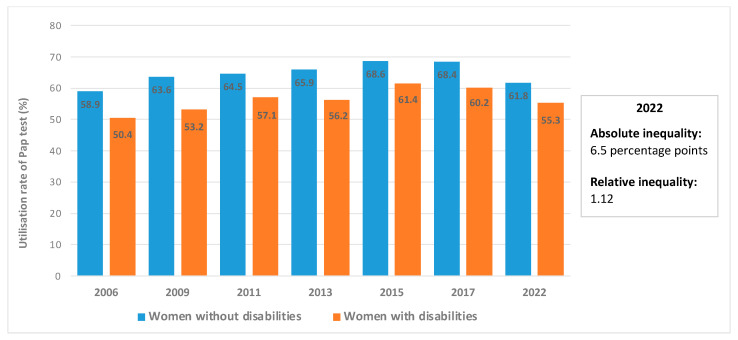
Utilisation rates of the Papanicolaou test by women with and without disabilities, 2006–2022. Note 1: All differences are statistically significant. Note 2: Due to the COVID-19 pandemic, the 2020 CASEN survey was abbreviated, and the questions on disability and Pap test were not included. Note 3: Absolute inequality is the difference between the percentage of women without disabilities and the percentage of women with disabilities who undertook the Pap test. Relative inequality is the quotient between these two percentages. Source: own elaboration with data from the Ministry of Social Development and Family [44,51,52,53,54,55,56].

Figure 1 presents Pap test utilisation by women with and without disabilities in Chile based on the various rounds of the CASEN surveys. The question on the realisation of the Pap test was included for the first time in the 2006 CASEN survey. As can be seen, there is a higher percentage of women without disabilities that undertake the Pap test in comparison to women with disabilities, with the largest absolute inequality observed in 2009 (10.4 percentage points) and the least observed in 2022 (6.5 percentage points). As a result of the COVID-19 pandemic, utilisation rates dropped for both groups. If we compare the pre- and post-pandemic rates, for women with disabilities, the utilisation rates dropped from 60.2% in 2017 to 55.3% in 2022. Meanwhile, for women without disabilities, utilisation rates went from 68.4% to 61.8%. The relative inequality in 2022 was 1.12, meaning that utilisation of the Pap test by women without disabilities is 1.12 times higher than Pap test utilisation by women with disabilities.

Simple and multivariable logistic regressions were performed to examine whether there is a difference in the probability of Pap test uptake between women with and without disabilities (Objective 1). Table 2 presents the results of the logistic regression and shows that women with disabilities had 18% lower odds of undertaking a Pap test compared to women without disabilities (OR: 0.820, 95% CI 0.771–0.871).

Table 3 presents the results of the multivariable logistic regression for women with disabilities regarding the characteristics associated with the realisation of the Pap test (Objective 2). The model includes demographic, socioeconomic, and health-related variables. With regards to the variables used, on the one hand, these are common variables used in research on access to healthcare, and on the other hand, they were available in the CASEN survey. Interaction terms were also employed. However, these were statistically insignificant and not included in the model.

The results in Table 3 show that women with disabilities in the 35–49 age group had 8.3 more odds of taking the Pap test in comparison to women from the 18–24 age group (OR: 8.259, 95% CI 6.056–11.263). Single women with disabilities were less likely to take the test compared to married women (OR: 0.609, 95% CI 0.524–0.708). Women who lived in a rural setting were more likely to undergo a Pap test (OR: 1.328, 95% CI 1.168–1.509).

In relation to education level, women with disabilities who only received special education had less than half the odds of taking the Pap test, compared to the group that had not attended school (OR: 0.401, 95% CI 0.248–0.648). On the contrary, women who had completed secondary or tertiary education had almost twice the odds of undergoing a Pap test compared to the group that had not attended school (OR: 1.859, 95% CI 1.418–2.437 and OR: 1.836, 95% CI 1.361–2.478, respectively).

Regarding employment, women with disabilities who were inactive were less likely to take the screening test, compared to employed women (OR: 0.622, 95% CI 0.546–0.708). Women who were under medical treatment for a certain condition were more likely to have a Pap test compared to women who were not under medical treatment (OR: 1.432, 95% CI 1.255–1.633). No statistically significant differences were observed regarding per capita family income and type of health insurance.

**Figure 2:**
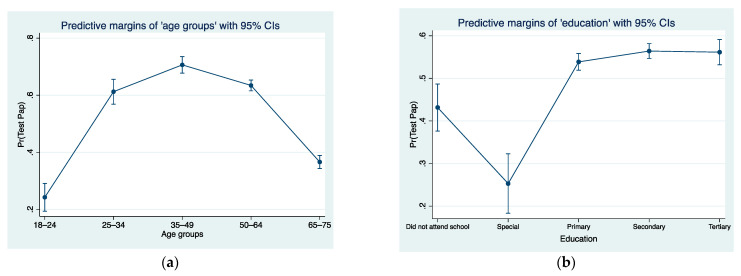
(**a**) Predictive probabilities for utilisation of Papanicolaou test with *age groups* as predictor variable. (**b**) Predictive probabilities for utilisation of Papanicolaou test with *education* as predictor variable.

Figure 2a presents the predicted probabilities for utilisation of the Papanicolaou test when the predictor variable is ‘age groups’. As it can be observed, women with disabilities in the 35–49 age group had the highest probability of undertaking a Pap test (70.6%), followed by women in the 50–64 age group (63.4%). With regards to Figure 2b, which shows the predictive probabilities for utilisation of the Papanicolaou test when the predictor variable is ‘education’, women with disabilities with secondary and tertiary education had the highest probability (56.4% and 56.1%, respectively) of undertaking a Pap test. The expected probability of a woman with disabilities who attended special education schools to undergo a Pap test was 25.3%.

## 4. Discussion

Our findings indicate that 61.8% of women without disabilities and 55.3% of women with disabilities had undergone the Pap test in the 2020–2022 period, which is a significant reduction if compared to the pre-Covid rates from 2017 (Figure 1). In the United States, significant state-level differences have been reported in the use of Pap testing between women with and without disabilities during the COVID-19 pandemic. In 2018, the screening rate was 69.4% among women with disabilities compared to 81.5% among women without disabilities. In 2020, these figures were 66.1% and 81%, respectively [57]. Another study analysing population-based surveys in Belgium, which included 92,334 individuals registered as disabled between 2013 and 2015, found that 45% of women with disabilities had undergone a Pap test in the previous three years, compared to 60.7% of women without disabilities [58]. Our study also indicates that the relative inequality with regard to the realisation of the Pap test was 1.12 in 2022, which was in favour of women without disabilities.

The question regarding the Pap test refers to the previous three years, that is, “In the last three years, have you had a Papanicolaou test?” This is followed by a question regarding the year when women undertook this test (that is, in 2020, 2021, or 2022), it was possible to calculate the utilisation rates for each of these years. For both groups, there was a significant increase in utilisation rates in 2022, in comparison to the previous two years when the impact of the pandemic was much more severe, and many quarantines and other types of preventive measures were in place. Out of the 6007 women with disabilities who replied that, during the period 2020–2022 they had undertaken the Pap test, 21.9% had undertaken it in 2020, 29.9% in 2021, and 45% in 2022. Accordingly, for women without disabilities, these percentages were 27.1%, 27.9%, and 48.3%, respectively.

In our sample, there were more women without disabilities (76.7%) in the 25–64 age range (that is, the target age for undertaking the Pap test) than women with disabilities (65.2%). The difference, however, in utilisation rates has been maintained since 2006, with the absolute inequality ranging from 6.5 to 10.4 percentage points, and without a clear increasing or decreasing pattern. It could be inferred that other factors, such as the social determinants of health and the structural disadvantage that many women with disabilities face, are behind the existing inequity in preventive exams utilisation.

The study evidenced that, after the COVID-19 pandemic, women with disabilities continued to be less likely to have a Pap test compared to women without disabilities. A separate regression that was performed using pre-pandemic data obtained from the 2017 CASEN revealed that women with disabilities had 25% lower odds of having a Pap test (OR: 0.75, 95% CI: 0.694–0.812; the regression is not presented here but is available upon request). Other international studies have shown that women without disabilities are twice as likely to have a Pap test compared to women with intellectual and developmental disabilities [5] and women with severe disabilities [59]; similar results were found in other studies that included women with visual impairment and mental illnesses [6,60].

Concerning the demographic characteristics that may influence Pap test utilisation by women with disabilities, women in the target age range (25 to 64 years old), according to the Ministry of Health of the Government of Chile, were more likely to undertake the Pap test. The women with the highest probability of undertaking the exam were women in the 35–49 age group, followed by women in the 50–64 age group and women in the 25–34 age group. Women outside the recommended age for the test, that is, women in the 65–75 age group and particularly women in the 18–24 age group, had a much lower probability of undertaking the Pap test. Being married was associated with a higher likelihood of undergoing the exam while being single or widowed with a lower probability. This fact may be related to the social stability of married women, in terms of living in the same place for a long time, having more frequent check-ups with doctors that they know at health centres that they regularly visit, and having family and friend networks [61,62]. In this study, living in a rural area is a protective factor for the realisation of the Pap test, which is a similar result to other studies [26,63,64].

Regarding socioeconomic characteristics, 60.9% of the women with disabilities were inactive, a much higher percentage than women without disabilities (39.5%). This is directly linked to the fact that women with disabilities often experience structural disadvantages in the form of increased poverty, lower employment rates, higher informal employment rates, and lower education levels in comparison to the general population. These differences lead to lower health services utilisation, more unmet healthcare needs, and worse health outcomes for this population subgroup [26,65]. In our study, the regression analysis also showed that inactive women with disabilities had a lower probability of taking the Pap test. Concerning education level, our findings indicated that women with disabilities who had secondary and tertiary education were the most likely to undergo a Pap test. Higher education levels have been associated with higher rates of adherence to preventive health programmes for people with disabilities, a fact which is most likely linked to having more access to health education [66,67].

An interesting result is that there seems to be no association between income level and the undertaking of the Pap test. While previous studies support the idea that income, as a social determinant, has the greatest or among the greatest effects on access to health care for people with disabilities [68,69], in the case of Chile, financial constraints are not a barrier to the realisation of the Pap test. Indeed, only 1.3% of women with disabilities and 1.3% of women without disabilities who stated that they had not undergone cervical cancer screening argued that the reason was financial (Table S2, Supplementary Material). Previous studies carried out in Chile where women were asked why they did not take the Pap test indicate that ignorance about the importance of screening is the main factor influencing adherence; women do not mention economic barriers as an important barrier [70,71]. This may occur because the Pap test is one of the preventive tests included free of charge in the Preventive Medicine Exams pack for the beneficiaries of the public (FONASA) and private (ISAPREs) insurance systems, which cover approximately 94% of the population. This exam is free only if accessed by women between 24 and 65 years of age who are FONASA and ISAPREs affiliates and if they undertake it once every three years with specific providers [27,31]. The economic factor could become a barrier if women do not comply with these requirements [72,73].

In relation to health-related characteristics, women with disabilities who were undergoing medical treatment for another condition were more likely to undergo the Pap test, which may be an indication of how people become more health conscious after or during an illness. The literature has evidenced that chronic diseases positively affect people’s participation in preventive health check-ups [63,74].

Our study also showed that the type of health insurance is not associated with Pap test utilisation. The Chilean health system is mainly composed of the public health insurance fund (FONASA), where approximately 76% of the population is affiliated, and private health insurance institutions (ISAPREs), with which 18% of the population is affiliated. This system is not complimentary, and people are affiliated with either the public or the private insurance system. FONASA affiliates (which mainly include people of a lower socioeconomic status) have generally worse access to health care services in comparison to ISAPREs affiliates (who are usually of a higher socioeconomic status), with regards to service quality, price, and waiting times, a fact which has created a stratification of users of the health system and increased health inequities [36,75]. However, in our study, we did not find significant differences between women with disabilities who belonged to FONASA or ISAPREs regarding the realisation of the Pap test. There was no difference either for women paying out of pocket, a result contradictory to other studies that evidenced lower utilisation rates of preventive exams by people with disabilities if they had to pay out of pocket [38]. The main reason behind the fact that the type of health insurance does not influence Pap test utilisation is the inclusion of this test in the Preventive Medicine Exams pack, which is one of the currently 87 health issues and/or problems of the GES (Garantías Explícitas en Salud) universal health plan, which provides explicit healthcare guarantees in the form of access, financial protection, opportunity, and quality of healthcare to all affiliates of the public and private system.

The lower access of women with disabilities to preventive programmes can have a negative impact on equity in health. Health education and promotion are key for reducing the existing inequity in the utilisation rates of the Pap test. In our study, out of the women who answered that they had not undergone a Pap test in the previous three years and were within the screening target group (that is, 25–64 years of age), 47.1% of the women with disabilities and 44.5% of the women without disabilities mentioned that they did not know that they had to undergo it, they did not know this test, they did not believe they needed it, or that the test did not apply to them (Table S2, Supplementary Material). This may be the product of misinformation associated with educational barriers [76,77,78] and a lack of effective health education and health promotion strategies on behalf of the state and health authorities.

The COVID-19 pandemic has posed additional challenges to reducing cervical cancer incidence due to various factors, including interruptions in vaccination, screening, and treatment services, quarantines and border closures reducing the availability of supplies, and school closures that interrupted school human papillomavirus (HPV) programmes [20]. The pandemic has further underlined the need for resilient health systems and services that do not collapse in case of crisis and can continue providing accessible, timely, appropriate, and good quality healthcare services for all. While during the COVID-19 pandemic, utilisation rates of the Pap test dropped, it is important to reverse this trend for all women, considering that the current rate (63%)—and even the pre-pandemic rate (66% in 2017)—is below the 80% national coverage goal for cervical cancer screening.

Public health policies should take into account the needs of women with disabilities. Strategies, programmes, and initiatives should consider intersectoral measures, such as increasing accessibility (concerning transport, health facilities, and equipment), and improving communication (for example, the use of sign language and documents that are easy to read). The role of (bio)technology, both for the development of accessible environments, as well as for the improvement of screening and diagnostic tests, and therapies, is also key for the increase in current utilisation rates for all women, and for women with disabilities in particular, that face additional barriers in accessing preventive exams. It is also important that population data are available on the barriers impeding women with disabilities from accessing exams; more research is needed that will shed light on the current situation and on how structural disadvantage affects access and utilisation of preventive exams by this population subgroup.

Educational strategies focused on the prevention of cervical cancer should also be introduced and/or strengthened, since there is ample evidence that such interventions are effective in raising awareness among both women and men about the transmission of the human papillomavirus, cancer evolution, and the importance of screening, leading to an increase in their intention to undergo screening [79,80]. Health education methods, such as telephone calls, postcards sent by mail, consultation sessions, books, videos, and web tools, should also be provided in accessible formats and consider the different types of disability [81,82], in order to ensure equitable access to information.

Health professionals should be trained on disability issues and health care provision for people with disabilities—a type of training that is severely lacking at the national and international levels—so that they are able to provide adequate and inclusive care [81,83]. It is also important that tailored health care strategies are designed and implemented, both during emergencies (for example, pandemics and natural disasters) and routine health care, that respond to the needs of people with different types of disability and living in a range of settings (for instance, institutional care, congregate residential settings, household settings, and prison) [84]. The participation of people with disabilities and their representative organisations is crucial for the elaboration, implementation, and evaluation of disability-inclusive health initiatives.

One of the limitations of this study derives from its cross-sectional methodological design, which only allows for determining association and not causality between variables. In addition, disability in the CASEN surveys is self-reported, which could affect the validity and reliability of this data. On the other hand, since this study was based on the analysis of a secondary database, it was not possible to add other factors that could influence the realisation of the Pap test, such as, for example, fear of COVID-19 contagion. Regarding future research, it would be relevant to explore in more detail the barriers that women with disabilities experience in accessing the Pap test, through a mixed methodological design, that can shed more light on their experience, an aspect that cannot be fully assessed through quantitative analysis alone. Another suggestion would be the realisation of a longitudinal study, so as to identify possible changes that may occur over a period of time for the same individuals that may influence their decision to undertake the Pap test. Moreover, it is also important to further analyse how health systems themselves promote or impede equity regarding access to preventive exams, and to identify their strengths and shortcomings, bearing in mind the need to increase their resilience, especially in times of crisis, so that they are capable of absorbing shocks and speed up their recovery [85,86] without a large negative impact on the provision of health services. Nevertheless, despite these limitations, the study is important, as it analyses post-COVID-19 population data on the potential demographic, socioeconomic, and health-related factors that may influence the realisation of cervical cancer screening tests by women with disabilities that have historically been neglected and/or largely excluded from research. The findings of the study are important to consider for the implementation of effective public health policies that will promote the uptake of the Pap test by women with disabilities.

## 5. Conclusions

The Papanicolaou test is the most widely used diagnostic test for the early detection of cervical cancer, one of the most common cancers affecting women’s health worldwide. Its use, along with other strategies, such as the HPV test, HPV vaccination, and cervical cancer treatment, has shown that it can lead to a significant reduction in cervical cancer incidence and mortality. However, due to existing structural disadvantages and inequities in health systems, women with disabilities are less likely to undergo screening tests, such as the Pap test.

Our study showed differences in the utilisation of the Pap test between women with and without disabilities in Chile, with women with disabilities being less likely to undertake the test. During the COVID-19 pandemic, utilisation rates by both groups fell significantly, with utilisation picking up in 2022 but below 2017 rates. The study evidenced that, for women with disabilities, there is a positive association between Pap test utilisation and being married, being in the target age range for the test, living in a rural area, having a higher education level, and being under medical treatment.

These findings emphasise the need for identifying risk and protective factors that may influence the realisation of the Pap test by women with disabilities, so as to generate inclusive and intersectoral health initiatives and public policies that are aimed at increasing screening coverage. The goal of reducing the incidence of cervical cancer and the associated morbidity and mortality rates cannot be achieved without guaranteeing equitable access to preventive exams for all population groups, particularly for disadvantaged groups such as women with disabilities.

## Figures and Tables

**Table 1:** Characteristics of women with and without disabilities.

Variables	n = 71,989		
	Women with Disabilities (n = 11,041)	Women Without Disabilities (n = 60,948)	*p* Value
**Papanicolaou test**			
Yes	6007 (54.5%)	36,932 (61.0%)	*p* < 0.001
No	5034 (45.5%)	24,016 (39.0%)	
**Age groups**			
18–24	594 (5.9%)	8424 (13.4%)	*p* < 0.001
25–34	880 (9.5%)	12,460 (23.7%)	
35–49	1832 (19.8%)	16,610 (30.2%)	
50–64	4309 (35.9%)	16,115 (22.8%)	
65–75	3426 (28.9%)	7339 (10.0%)	
**Civil status**			
Married	3871 (35.4%)	18,761 (31.3%)	*p* < 0.001
Living with or in a relationship	1422 (14.4%)	12,800 (24.2%)	
Separated, divorced or annulled	1520 (13.6%)	5501 (8.3%)	
Widowed	1172 (9.4%)	2687 (3.6%)	
Single	3056 (27.2%)	21,199 (32.6%)	
**Zone**			
Urban	8928 (88.5%)	49,283 (89%)	*p* = 0.024
Rural	2113 (11.5%)	11,665 (10.7%)	
**Education**			
Did not attend school	414 (3.9%)	448 (0.5%)	*p* < 0.001
Special	251 (2.3%)	54 (0.09%)	
Primary	4028 (31.5%)	11,126 (14.0%)	
Secondary	4285 (39.5%)	26,725 (41.7%)	
Tertiary	2063 (23.9%)	22,595 (43.7%)	
**Employment**			
Employed	3415 (34.9%)	30,678 (54.5%)	*p* < 0.001
Unemployed	427 (4.2%)	3467 (6.0%)	
Inactive	7199 (60.9%)	26,803 (39.5%)	
Per capita family income *			
0–350,000	5735 (48.4%)	31,628 (47.2%)	*p* < 0.001
350,001–500,000	2660 (23.7%)	12,229 (19.7%)	
500,001–1,000,000	2185 (21.5%)	12,499 (22.0%)	
1,000,001+	461 (6.4%)	4592 (11.1%)	
**Health Insurance**			
FONASA (Public)	10,126 (88.5%)	52,448 (80.8%)	*p* < 0.001
ISAPRE (Private)	563 (8.3%)	5917 (14.8%)	
Armed Forces and Order	135 (1.4%)	797 (1.3%)	
None (Out-of-Pocket)	184 (1.9%)	1557 (3.2%)	
**Medical Treatment**			
No	2826 (27.8%)	38,242 (65.7%)	*p* < 0.001
Yes	8105 (72.1%)	21,894 (34.3%)	

* Income is presented in Chilean pesos (1 USD = 872.33 CHP, 2022 average).

**Table 2:** Logistic regressions on the utilisation of the Papanicolaou test by women with and without disabilities.

	OR	95% CI	Obs.	Sub-Population Size	F (*p* Value)
Simple logistic regression					
Women with disabilities	0.765	0.726–0.807	71,989	6,746,130	96.66 (*p* < 0.001)
Multivariable logistic regression *					
Women with disabilities	0.820	0.771–0.871	71,067	6,658,856	239.13 (*p* < 0.001)

OR: odds ratio; 95% CI: 95% confidence interval; Obs.: observations; F (*p* value): F statistic, *p* value. * Adjusted for age, civil status, zone, education, employment, per capita family income, health insurance, and medical treatment.

**Table 3:** Multivariable logistic regression analysis on the characteristics associated with the utilisation of the Papanicolaou test by women with disabilities.

	Coefficient	Std. Error	OR	95% CI	*p* Value
Age groups (Reference: 18–24)					
25–34	1.668	0.156	5.302	3.906–7.199	*p* < 0.001
35–49	2.111	0.158	8.259	6.056–11.263	*p* < 0.001
50–64	1.766	0.150	5.850	4.361–7.847	*p* < 0.001
65–75	0.612	0.156	1.845	1.358–2.506	*p* < 0.001
Civil status (Reference: Married)					
Living with or in a relationship	0.140	0.091	1.150	0.962–1.374	*p* = 0.125
Separated, divorced, annulled	−0.166	0.088	0.847	0.713–1.006	*p* = 0.058
Widowed	−0.297	0.093	0.743	0.620–0.891	*p* = 0.001
Single	−0.496	0.077	0.609	0.524–0.708	*p* < 0.001
Zone (Reference: Urban)					
Rural	0.284	0.065	1.328	1.168–1.509	*p* < 0.001
Education (Reference: Did not attend school)					
Special	−0.913	0.244	0.401	0.248–0.648	*p* = 0.004
Primary	0.500	0.136	1.649	1.264–2.152	*p* < 0.001
Secondary	0.620	0.138	1.859	1.418–2.437	*p* < 0.001
Tertiary	0.608	0.153	1.836	1.361–2.478	*p* < 0.001
Employment (Reference: Employed)					
Unemployed	−0.029	0.147	0.971	0.728–1.295	*p* = 0.842
Inactive	−0.475	0.066	0.622	0.546–0.708	*p* < 0.001
Per capita family income (Reference: 0–350,000 CHP)					
350,001–500,000	0.049	0.064	1.050	0.927–1.190	*p* = 0.442
500,001–1,000,000	−0.028	0.076	0.973	0.837–1.130	*p* = 0.718
1,000,001+	−0.216	0.197	0.806	0.548–1.184	*p* = 0.272
Health insurance (Reference: FONASA)					
ISAPRE (private)	0.283	0.185	1.327	0.924–1.905	*p* = 0.126
Armed Forces and Order	0.216	0.231	1.242	0.790–1.953	*p* = 0.349
None (out-of-pocket)	−0.190	0.228	0.827	0.529–1.294	*p* = 0.406
Medical treatment (Reference: No)					
Yes	0.359	0.067	1.432	1.255–1.633	*p* < 0.001
Observations	10,931				
Sub-population size	902,130				
F	44.32				
*p*	*p* < 0.001				

## Data Availability

The datasets analysed during the current study are available in the repository “Social Observatory” of the Ministry of Social Development and Family of the Government of Chile. “URL: https://observatorio.ministeriodesarrollosocial.gob.cl/encuesta-casen-2022 (accessed on 5 August 2024)”. To access the database, go to “Bases de datos”. From there, you can download the database in Stata or SPSS.

## Supplementary Materials

The following supporting information can be downloaded at https://www.mdpi.com/article/10.3390/ijerph21121578/s1: Table S1. STROBE Statement—Checklist of items that should be included in reports of cross-sectional studies; Table S2. Reasons for not undergoing the Pap test for women with and without disabilities.
